# Influence of Selective Agents (EMJH-STAFF), Sample Filtration and pH on *Leptospira interrogans* Serovar Icterohaemorrhagiae Cultivation and Isolation from Swine Urine

**DOI:** 10.3390/vetsci8060090

**Published:** 2021-05-25

**Authors:** Romana Steinparzer, Tamara Mair, Christine Unterweger, Adi Steinrigl, Friedrich Schmoll

**Affiliations:** 1 Institute for Veterinary Disease Control, Austrian Agency for Health and Food Safety (AGES), Robert Koch Gasse 17, 2340 Mödling, Austria; adi.steinrigl@ages.at (A.S.); Friedrich.Schmoll@ages.at (F.S.); 2 University Clinic for Swine, Department for Farm Animals and Veterinary Public Health, University of Veterinary Medicine Vienna, Veterinaerplatz 1, 1210 Vienna, Austria; tamara.mair@aon.at (T.M.); christine.unterweger@vetmeduni.ac.at (C.U.)

**Keywords:** *Leptospira*, Icterohaemorrhagiae, swine urine, EMJH-STAFF, isolation, pH, PBS

## Abstract

*Leptospira* spp. cause the zoonotic disease leptospirosis, which occurs in numerous mammalians worldwide. Isolation is still important for serotyping and genotyping of *Leptospira*, which in turn is essential for epidemiological surveillance of leptospirosis and the development of diagnostic tests and vaccines. However, isolation of *Leptospira* from clinical specimens is inherently insensitive. This study was conducted to examine the influence of selective agents, sample filtration, sample pH and the use of phosphate buffered saline (PBS) buffer for sample storage to improve the success of cultivation and isolation of *Leptospira interrogans* serovar Icterohaemorrhagiae from swine urine. EMJH (Ellinghausen McCullough, Johnson and Harris) medium including the selective agents sulfamethoxazole, trimethoprim, amphotericin, fosfomycin and 5-fluorouracil (STAFF) increased the success of *Leptospira* isolation from spiked swine urine samples. Sample filtration yielded only negative results. Isolation in EMJH-STAFF was successful from swine urine with a density as low as 10^4^ *Leptospira*/mL, and urine with pH ≤ 7 impaired the cultivation rate. Cultivation and isolation were not improved by the addition of PBS to spiked urine samples prior to storage for 24 h at 4 °C. The results of the study demonstrate that cultivation and isolation of leptospires from swine urine can be improved by enhanced methods.

## 1. Introduction

Leptospirosis is a worldwide occurring zoonotic disease caused by pathogenic *Leptospira* species. Numerous mammals are susceptible to the spirochetal disease including livestock and humans. In sows, leptospirosis causes infertility, abortion and stillbirth [1].

Direct detection of *Leptospira* from samples can be performed by molecular diagnostic techniques and cultivation. *Leptospira* mainly affect the kidneys, the urogenital tract and the liver and they are shed in urine [2]. Thus, tissue samples from affected organs and urine can be used for direct *Leptospira* detection. Molecular techniques are usually able to provide results within a short period of time, are (semi-)quantitative in the case of quantitative PCR (qPCR), less laborious to perform and several studies are available for samples of animal origin [3,4,5,6,7,8,9,10]. In comparison, cultivation can take from a few days to several months depending on the serovar and requires well-equipped laboratories. Furthermore, *Leptospira* cultivation and further isolation is characterized by low sensitivity and improvement of the technique is needed [11], in particular because *Leptospira* isolation is a prerequisite for serological and important for molecular *Leptospira* characterization, such as whole genome sequencing. Methods for genotyping *Leptospira* directly from extracted DNA of clinical samples without the need for isolation of *Leptospira* became available in the past years [12]. The characterization of circulating *Leptospira* within a host species and region is essential for epidemiological surveillance of leptospirosis and the development of diagnostic tests and vaccines. For swine, no current and only a few older studies on successful isolation of *Leptospira* are available. Isolation from swine tissue samples of *Leptospira interrogans* serogroup Icterohaemorrhagiae and Hebdomadis in the south of England from swine kidneys [13], serogroup Icterohaemorrhagiae, Hebdomadis, Australis and Autumnalis from aborted fetuses in Northern Ireland [14], serovar Bratislava from a swine kidney in Germany [15], serovar Bratislava and Hardjo from the kidneys and genital tracts of swine in Iowa, USA [16], serovar Grippotyphosa and Kennewicki from aborted fetuses in Iowa, USA [17] and serovar Pomona from swine genital tracts, livers and kidneys in Brazil [18] was reported. *Leptospira interrogans* serogroup Australis was isolated from the urine of one pig in Brazil [19].

Different media are described for *Leptospira* cultivation and isolation and one of the most common is the Ellinghausen McCullough, Johnson and Harris (EMJH) medium [2,20]. Various selective agents are recommended to be added to media particularly for contaminated sample material [2]. Since clinical samples from animals are mostly taken under field conditions, contamination occurs frequently. A combination of the selective agents 5-fluorouracil, chloramphenicol, nalidixic acid, neomycin and vancomycin was used to isolate *Leptospira* from cattle urine [21]. Sulfamethoxazole, trimethoprim, amphotericin, fosfomycin and 5-fluorouracil, abbreviated as STAFF, were successfully used to isolate pathogenic *Leptospira* from environmental soil and water [22]. The same combination of substances was confirmed to be effective for contamination control during cultivation of *Leptospira* from cattle urine and vaginal fluid [23]. Another way to reduce contamination is by filtration of processed sample material [2]. Successful isolation of *Leptospira* from urine is challenging [24,25] and whilst temperature and pH were described to affect *Leptospira* viability in water [26,27], little is known about urine.

Isolation and characterization of circulating *Leptospira* is also essential for the use of sufficient serovars in the microscopic agglutination test (MAT) for antibody detection. The MAT is the gold standard according to the World Organisation for Animal Health (OIE) and is the predominantly used technique for serological diagnosis. A pure culture of a *Leptospira* serovar is used in the MAT to identify antibodies against that serovar. Worldwide, more than 300 serovars are known. Due to the high number of existing serovars, the number of tested serovars in the MAT may depend on the capability of the lab. It is recommended to use serovars or at least serogroups that are known to be prevalent within a species and region to prevent false negative results [28,29,30]. Constant isolation and serotyping of circulating *Leptospira* is therefore required. Furthermore, a limitation of the MAT is that antibodies can only be differentiated according to serogroups but not serovars. This is due to the cross-reactivity of antibodies against serovars belonging to the same serogroup. Identification of a serovar is solely possible from *Leptospira* isolates through typing by either serological [2] or molecular techniques [31] and then again, *Leptospira* isolation is a prerequisite. 

Data about seropositivity in swine are available from different regions and time intervals and for selected *Leptospira* serovars. Within European countries the mean seropositivity in France was 26.5% between 1988 and 2007 [32], in north central Italy 19.7% between 2002 and 2016 [33], in Italy 18.6% between 2010 and 2011 [34] and in Germany 20.2% between 2011 and 2016 [35]. Further studies report seroprevalences of 10.0% in central Thailand in 2004 and 2005 [36], 21.1% in Vietnam (10 selected provinces) for 2017 [37], 64.8% in Saint Kitts (Caribbean region) for 2016 and 2017 [38] and 32.9% in Kenya for 2018 [39]. The serological data show the occurrence of leptospiral infections all over the world, however limited success of *Leptospira* cultivation and isolation is described, which might depend, besides other reasons such as antibiotic treatments in intensive farming systems, on the cultivation technique used and lack of standardization. 

The aim of our study was to determine the influence of urine pH, selective agents (STAFF) and sample filtration on the success of *Leptospira interrogans* serovar Icterohaemorrhagiae cultivation and isolation from spiked swine urine. Furthermore, the study aimed to define the required *Leptospira* minimum concentration in urine for successful cultivation. Cultures were examined for leptospiral growth by both microscopic assessment and resource-saving molecular quantification by qPCR. The effect on cultivation and isolation from urine storage and the effectivity of using phosphate buffered saline (PBS) buffer to neutralize pH and dilute the urine was also tested.

## 2. Materials and Methods

### 2.1. Sample Collection and Leptospira Strain

Non-invasive samples of urine (voided urine) were taken from 30 sows from 2 Austrian farms. Thirteen samples originated from the first farm and 17 from the second one. The samples were stored at −20 °C. The following study was divided into three separate experiments. In all three experiments, a pure culture of *Leptospira interrogans* serovar Icterohaemorrhagiae strain RGA (purchased from Academic Medical Centre, Leptospirosis Reference Centre, Amsterdam, Netherlands) was used to spike the urine samples. Before each experiment the culture was tested on blood agar to exclude bacterial contamination. Serovar Icterohaemorrhagiae was chosen due to its relevant pathogenicity in swine [2,35,37], worldwide occurrence [40] and relatively fast growth [2]. All urine samples were tested with qPCR as described in experiment 1 before spiking to exclude any existing contamination with *Leptospira*. In the experiments, two non-inoculated controls to identify any medium contamination and two controls using medium instead of urine to verify *Leptospira* viability and growth were incorporated. 

### 2.2. Experiment 1—Selective Agents in Culture Medium, Sample Filtration and qPCR Quantification

**Figure 1:**
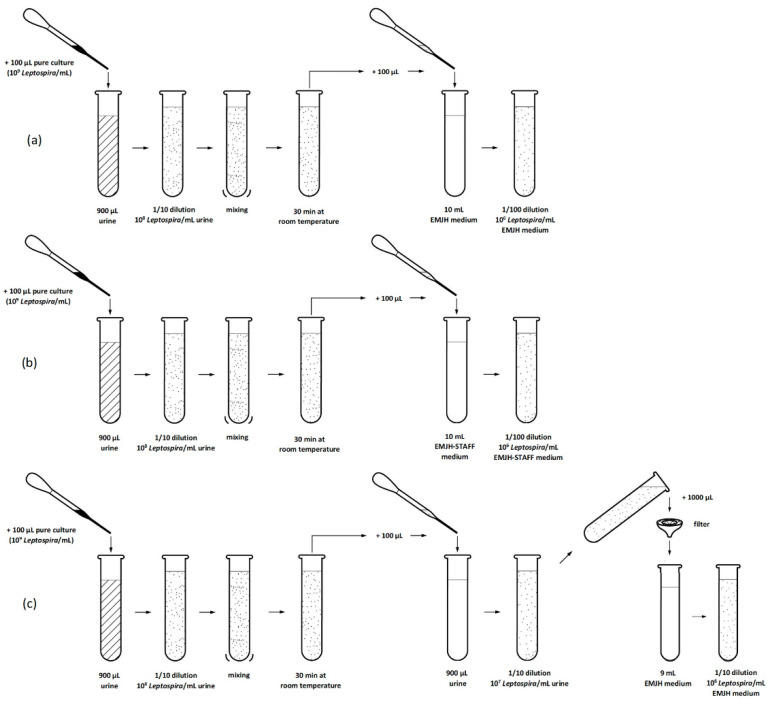
Experiment 1: Process of culture preparation (**a**) in EMJH medium, (**b**) in EMJH-STAFF medium and (**c**) with filtration of each urine sample.

Selective agents (STAFF) in culture medium and sample filtration were used to reduce contamination and support growth and isolation of *Leptospira* from swine urine samples with different pH values. The stored urine samples were thawed at room temperature and the pH and temperature were measured. A Helber Counting Chamber was used to determine the density (*Leptospira*/mL) of a *Leptospira interrogans* serovar Icterohaemorrhagiae pure culture as described elsewhere [30]. Liquid EMJH medium (DifcoTM Leptospira Medium Base EMJH (batch: 5112596) and *Leptospira* Enrichment EMJH (batch: 8253901 and 8360783), Becton Dickinson) was prepared according to the manufacturer’s instructions. The EMJH medium was divided into two portions. STAFF (sulfamethoxazole, trimethoprim, amphotericin, fosfomycin and 5-fluorouracil) was added into one portion to receive a final concentration of 40 µg sulfamethoxazole, 20 µg trimethoprim, 5 µg amphotericin, 200 µg fosfomycin and 100 µg 5-fluorouracil in one mL EMJH medium. Pure culture with a density of 10^9^ *Leptospira*/mL was used. Three 1/10 dilutions of pure culture in urine were prepared for each of the 30 urine samples and left at room temperature for 30 min. One hundred microliters of two dilutions was added to either 10 mL EMJH or 10 mL EMJH-STAFF medium. One hundred microliters of the remaining dilution was mixed with 900 µL of the unspiked urine and then passed through a filter (32 mm diameter with a membrane of 0.8/0.2 μm pore size) into 9 mL EMJH medium. Therefore, we obtained for each of the 30 urine samples an EMJH culture, an EMJH-STAFF culture and an EMJH culture after filtration each containing 10^6^ *Leptospira*/mL (Figure 1). The cultures were incubated at 29 °C and the remaining urine samples were again stored at −20 °C. In total, we had 90 cultures that were evaluated for *Leptospira* and contamination under dark field microscope (200×) every seven days for four weeks. A standardized scheme was defined for microscopic evaluation. The number of *Leptospira* were counted in five fields of view in five horizontal lines. The average amount of *Leptospira* counted in a field of view was calculated and assigned to one of four categories (category 0: no *Leptospira*, category 1: 1–5 *Leptospira*, category 2: 5–100 *Leptospira*, category 3: >100 *Leptospira*). The cultures were also differentiated into four other categories regarding the microscopic detection of *Leptospira* and contaminants (no *Leptospira*/no contamination, contamination, *Leptospira* plus contamination, pure *Leptospira*).

Quantitative PCR (qPCR), targeting the leptospiral outer membrane lipoprotein gene *lipL32* was used to detect and quantify *Leptospira* growth in culture. Each of the 90 cultures was sampled twice, at day two and 28 days after inoculation. One microliter of each culture was heated to 95 °C for 15 min and then stored at −20 °C until qPCR analysis. For the analysis 5 µL of heat-treated bacterial cultures were directly subjected to qPCR without a separate nucleic acid extraction step. Reaction mixes consisted of each 0.3 µM of primers LipL32_412F (5′-GAA AGA ATG TCG GCG ATT ATG C-3′) as well as LipL32_485Rmod (5′-TCG TYC AAT TTT TGA ACK GGT TT-3′) and 0.2 µM of the fluorescent probe LipL32_438probe-FAM (5′FAM-CCAAATCGCCAAAGCTGCGAAAGC-3′BHQ1) in 25 µL reaction volume (QuantiTect Multiplex PCR NoROX Kit, Qiagen, Hilden, Germany). After an initial denaturation step of 95 °C/5 min, qPCR was performed for 45 cycles of 94 °C/1 min and 60 °C/1 min on a CFX96 Touch Real-Time PCR Detection System (Biorad, Hercules, CA, USA). Absence of leptospires in urine samples before spiking was confirmed with the same qPCR protocol, but followed by nucleic acid extraction with the QIAamp Viral RNA Mini Kit (Qiagen, Germany) to get rid of potential PCR inhibitors present in urine. Quantification of leptospiral load in cultures was done by parallel amplification of a ten-fold dilution series of *Leptospira interrogans* serovar Icterohaemorrhagiae genomic DNA standard. The latter was prepared by extraction of a pure culture with known density (10^9^ *Leptospira*/mL, as determined with a counting chamber) using the MagAttract HMW DNA Mini Kit (Qiagen, Hilden, Germany). Leptospiral genome equivalents (expressed as copies/mL) in the standard were determined both by spectrophotometry (DeNovix DS-11 FX spectrophotometer/fluorometer, Biozym, Wien, Austria) and fluorometry (Qubit 4 Fluorometer, Thermo Fisher Scientific, Waltham, MA, USA). 

### 2.3. Experiment 2—Leptospira Dilution Series

**Figure 2:**
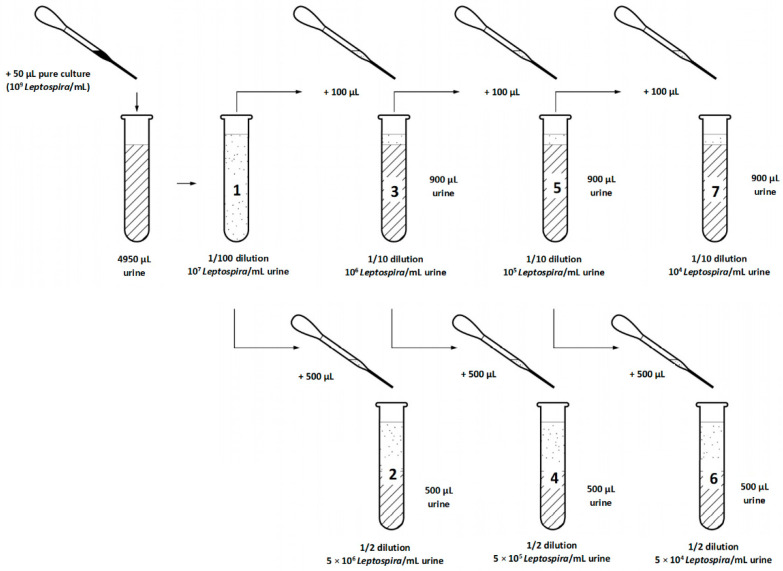
Experiment 2: Steps of preparation of seven dilutions of pure culture (10^9^ *Leptospira*/mL) in each of ten selected urine samples before transfer into culture medium (EMJH-STAFF).

Dilution series were used to define the minimum required *Leptospira* concentration in urine for cultivation. The urine samples were thawed at room temperature and the pH and temperature were measured. The density of a *Leptospira interrogans* serovar Icterohaemorrhagiae pure culture was determined as in experiment 1. EMJH-STAFF medium was prepared and ten urine samples were selected based on successful *Leptospira* detection and low contamination in experiment 1. Seven dilutions (1 × 10^7^, 5 × 10^6^, 1 × 10^6^, 5 × 10^5^, 1 × 10^5^, 5 × 10^4^ and 1 × 10^4^ *Leptospira*/mL) of pure culture (10^9^ *Leptospira*/mL) in urine were prepared for each of the ten urine samples and left at room temperature for 30 min (Figure 2). One hundred microliters of each dilution was added to 10 mL EMJH-STAFF. Therefore, we obtained for each of the ten urine samples seven EMJH-STAFF cultures containing 1 × 10^5^, 5 × 10^4^, 1 × 10^4^, 5 × 10^3^, 1 × 10^3^, 5 × 10^2^ and 1 × 10^2^ *Leptospira*/mL. The remaining urine samples were again stored at −20 °C. In total, we had 70 cultures that were incubated and evaluated microscopically as in experiment 1 every seven days but for seven weeks. 

### 2.4. Experiment 3—PBS Buffer for Sample Storage

PBS buffer was added to the spiked urine samples prior to storage to simulate sample transport to the laboratory. PBS buffer was used to neutralize urine pH and dilute the urine to support viability and isolation of *Leptospira*. The urine samples were thawed at room temperature and the pH and temperature were measured. The density of a *Leptospira interrogans* serovar Icterohaemorrhagiae pure culture was determined as described in experiment 1. EMJH, EMJH-STAFF medium and phosphate buffered saline (PBS) buffer (8.50 g NaCl, 0.49 g KH_2_PO_4_, 1.14 g Na_2_HPO_4_ 2H_2_O in 1000 mL Aqua bidest; pH 7.3) were prepared. Ten urine samples were selected in order to represent a wide pH range. A 1/10 dilution of pure culture (10^9^ *Leptospira*/mL) in urine was prepared for each of the ten urine samples. The dilutions were divided into two aliquots and the same quantity PBS was added to one of them. PBS was used for urine sample dilution and to approximate the pH closer to a slight alkaline value. Dilutions with and without buffer were stored at 4 °C for 24 h. After the storage period, 100 µL of each dilution was added to 10 mL EMJH and 10 mL EMJH-STAFF. The remaining urine samples were again stored at −20 °C. In total, we had 40 cultures that were incubated and evaluated microscopically as performed in experiment 1 every seven days over a four week period.

### 2.5. Calculation and Statistical Analysis

Microsoft Excel^®^ 2016 (Microsoft Corporation, Redmond, WA, USA) was used for data documentation and calculations. Chi-squared test was used to determine significant relations (*p* < 0.01) and Spearman correlation coefficient to determine correlations. *t*-test was used to calculate significant differences between observed leptospiral copy numbers by qPCR.

## 3. Results

All 30 urine samples tested negative for *Leptospira* before spiking and usage in the three experiments.

### 3.1. Experiment 1—Influence of Selective Agents in Culture Medium, Sample Filtration and Urine pH on Leptospiral Growth

**Figure 3:**
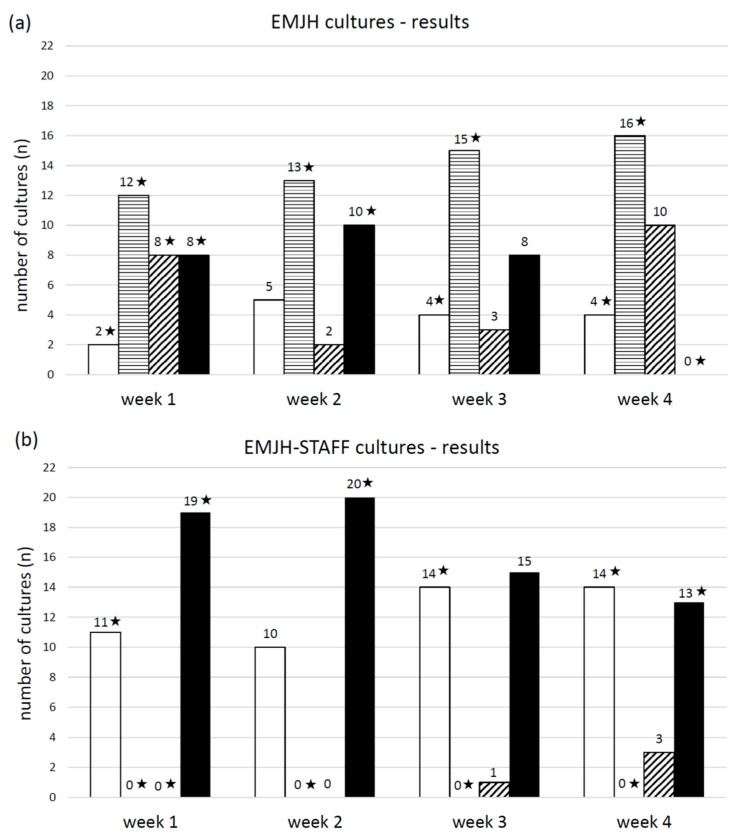
Number of (**a**) EMJH and (**b**) EMJH-STAFF cultures from spiked swine urine samples with the microscopic (dark field 200×) evaluation results at week one, two, three and four post inoculation differentiated into the following categories: □ no Leptospira/no contamination, ▤ contamination, ▨ Leptospira plus contamination and ■ pure Leptospira. ★ significant relationship between the result category at the corresponding week of evaluation and culture (EMJH and EMJH-STAFF), (*p* < 0.01).

**Figure 4:**
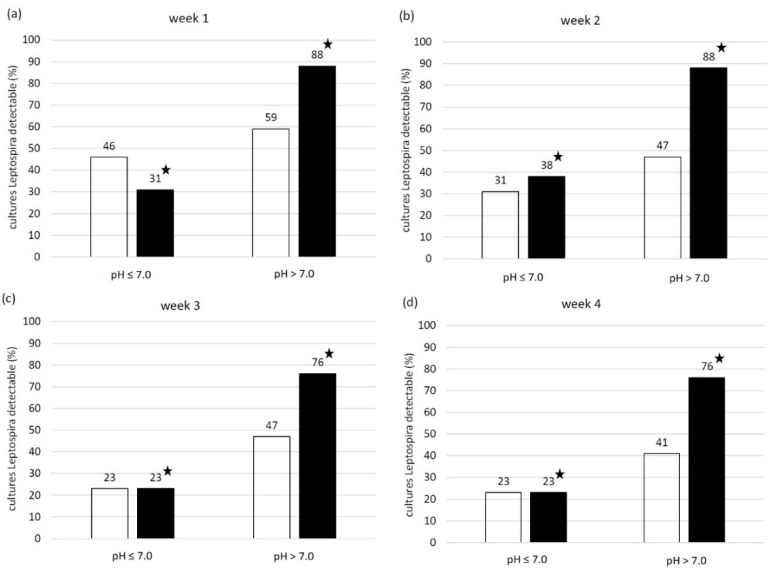
Percentage of □ EMJH and ■ EMJH-STAFF cultures from spiked swine urine samples with pH ≤ 7 and > 7 and microscopically (dark field, 200×) detectable Leptospira with and without contamination at (**a**) week one, (**b**) week two, (**c**) week three and (**d**) week four post inoculation. ★ significant relationship between urine sample pH and detectability of Leptospira in cultures at the corresponding week of evaluation (*p* < 0.01).

The pH of the 30 urine samples ranged from 6.4 to 8.5 and the mean temperature was 21.0 °C. The number of samples with a pH ≤ 7.0 was 13 and with a pH > 7.0 was 17. EMJH cultures of all four categories (no Leptospira/no contamination, contamination, Leptospira plus contamination, pure Leptospira) were identified. One week post inoculation, leptospiral growth was observed by dark-field microscopy in 16 EMJH cultures, of which 8 cultures also showed contamination. Four weeks post inoculation only leptospiral growth with contamination was seen in 10 EMJH cultures. In comparison to EMJH cultures, the number of EMJH-STAFF cultures with leptospiral growth was higher every week and all of them—except one culture in week three and three cultures in week four—were free from contamination. Most cultures with Leptospira plus contamination were detected after four weeks and with pure Leptospira after two weeks in both EMJH and EMJH + STAFF media. Contaminated EMJH-STAFF cultures without leptospiral growth were not observed but more EMJH-STAFF cultures were without Leptospira and contamination compared to EMJH cultures (Figure 3). Sixteen EMJH cultures had to be discarded after two weeks due to the high degree of contamination. The discarded samples were subsequently assigned to the category of cultures with only contamination in week three and four. None of the cultures with filtered samples showed any growth of Leptospira or contamination (data not shown). Every week the percentage of cultures with leptospiral growth (with and without contamination) was higher from samples with a pH > 7 than with a pH ≤ 7 using either EMJH or EMJH-STAFF medium. Significant differences were shown for EMJH-STAFF cultures (*p* < 0.01) (Figure 4). 

**Figure 5:**
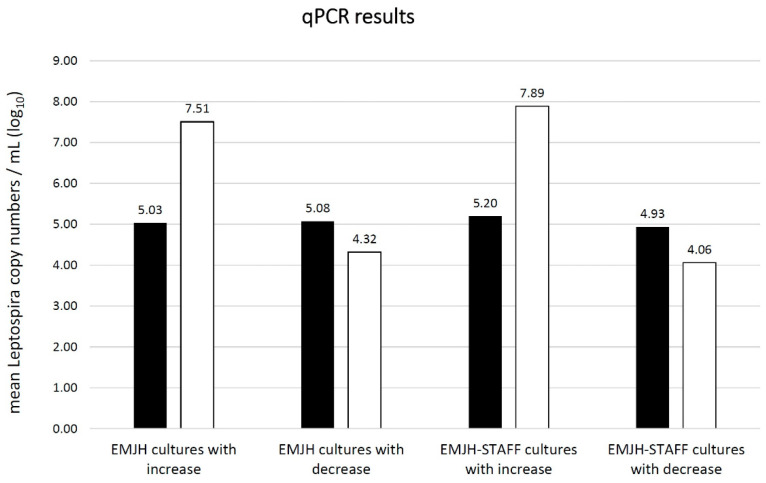
Mean absolute Leptospira copy numbers per mL (log_10_) as estimated by qPCR in EMJH and EMJH-STAFF cultures with Leptospira copy number increase and decrease ■ 2 days (n = 30 EMJH cultures; n = 30 EMJH-STAFF cultures) compared to □ 28 days (n = 14 EMJH cultures; n = 30 EMJH-STAFF cultures) post inoculation.

In both EMJH and EMJH-STAFF cultures, the mean copy number of Leptospira at day two after inoculation was around 10^5^ copies/mL (Figure 5). At this point in time, there was no significant difference (*p* = 0.09) in the copy numbers observed between EMJH and EMJH-STAFF cultures. In ten EMJH and 15 EMJH-STAFF cultures a decrease of Leptospira copies/mL was seen, while an increase was seen in four EMJH and fifteen EMJH-STAFF cultures 28 days after inoculation. All cultures with a copy-number increase belonged to the microscopically determined categories 2 or 3, with the exception of one EMJH-STAFF culture assigned to category 1. All cultures with a copy-number decrease belonged to category 0 in microscopic evaluation with the exception of one EMJH-STAFF culture assigned to category 1. Cultures that showed Leptospira increase at 28 days after inoculation reached genome copy numbers of >10^7^ copies/mL, whereas cultures with Leptospira decrease remained clearly below 10^5^ copies/mL (Figure 5). EMJH-STAFF cultures reached significantly higher copy numbers after 28 days than EMJH cultures (*p* < 0.01). The correlation coefficient between the microscopically determined categories and those determined by qPCR was 0.72 for EMJH, 0.84 for EMJH-STAFF and 0.84 for EMJH together with EMJH-STAFF cultures. 

In the filtrated samples, the mean genome copy number as determined by qPCR was below the threshold for quantification of 10^3^ copies/mL at both tested time-points (data not shown). 

### 3.2. Experiment 2—Influence of Leptospira Concentration in Urine on Their Growth in Culture Medium

The pH of the ten urine samples ranged from 7.2 to 8.3 and the mean temperature was 21.3 °C. The limit of Leptospira detection from urine samples was 1 × 10^4^ Leptospira/mL (1 × 10^2^ Leptospira/mL in culture). More cultures with a high Leptospira density were without contamination at the early stage of culture. After six weeks contaminants were microscopically detected in cultures with a high Leptospira density and the percentage of cultures with Leptospira and without contamination decreased (Table 1). 

### 3.3. Experiment 3—Influence of PBS Buffer Added for Sample Storage on Leptospira Growth

**Figure 6:**
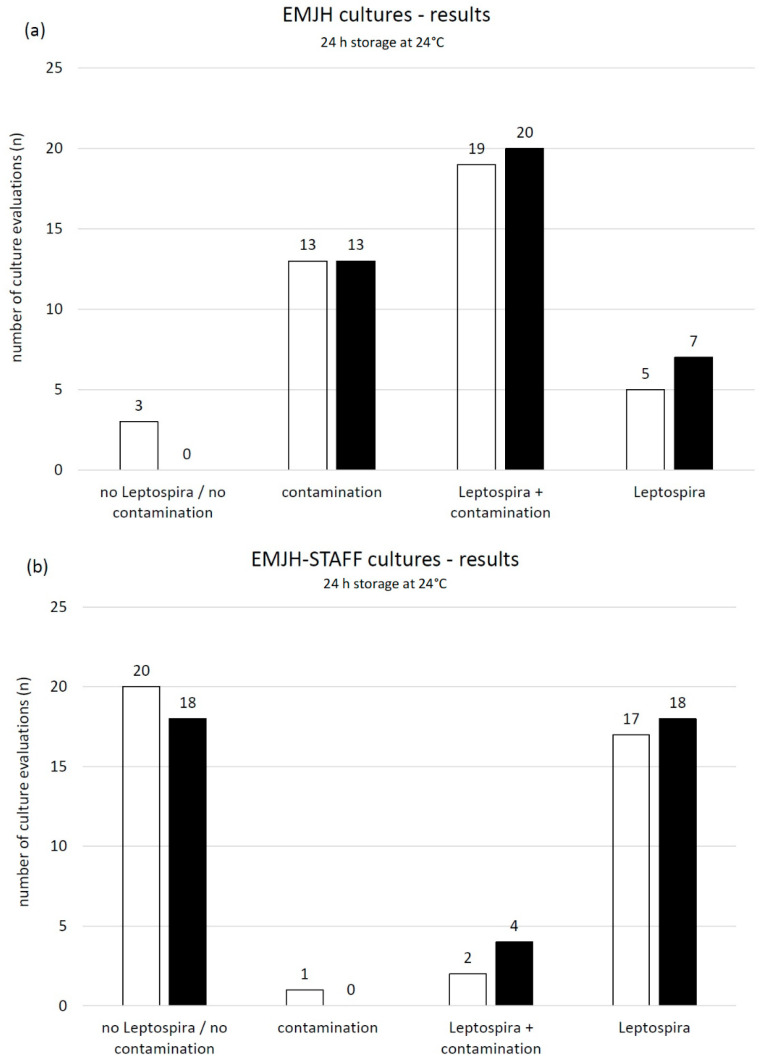
Number of microscopic (dark field, 200×) evaluations including week one, two, three and four post inoculation of (**a**) ten EMJH and (**b**) ten EMJH-STAFF cultures from spiked swine urine samples stored for 24 h at 4 °C □ without and ■ with added buffer (PBS) and differentiated into the following categories: no Leptospira/no contamination, contamination, Leptospira plus contamination and pure Leptospira.

The pH of the ten urine samples ranged between 6.4 and 8.4 and the mean temperature was 22.3 °C. The pH of samples with PBS changed to values between 6.9 and 7.9. The minimum change was 0.0 and the maximum was 0.6 closer to the buffer pH of 7.3. Evaluation results (no Leptospira/no contamination, contamination, Leptospira plus contamination, pure Leptospira) of cultures from samples with and without buffer stored at 4 °C for 24 h were compared using either EMJH or EMJH-STAFF medium. No significant relations were identified (Figure 6). 

## 4. Discussion

Leptospirosis mainly causes reproductive disorders in pigs with a negative impact on animal welfare and the economy of swine farming. 

*Leptospira* is shed by urine, which is therefore the specimen of choice for Leptospira isolation from live swine [41]. Sterile, non-invasive sampling of urine from swine is frequently not possible and contamination of urine samples is therefore highly likely. A urinary catheter can be used to obtain sterile urine samples, however it is a time consuming and invasive method that should be avoided for animal welfare reasons [42]. Various contaminants grow in *Leptospira* media, making it difficult to get pure *Leptospira* isolates. EMJH-STAFF medium contains selective agents to suppress the growth of contaminants. In the described study, the use of EMJH-STAFF medium was more successful in isolation of *Leptospira interrogans* serovar Icterohaemorrhagiae from swine urine compared to EMJH without selective agents. The result is comparable with studies using water and soil and cattle urine as sample material for *Leptospira* isolation with EMJH-STAFF medium [22,23]. EMJH-STAFF medium is therefore recommended for primary isolation of *Leptospira interrogans* serovar Icterohaemorrhagiae from swine urine. In some pure Leptospira cultures, contamination became microscopically visible in later evaluations in both EMJH and EMJH-STAFF cultures. In some further pure cultures, we were not able to visualize *Leptospira* microscopically in later evaluations and the assumption is that they died. A solution to prevent growth of present contaminants and to maintain *Leptospira* viability might be subcultivation as described in further studies [21,23]. Subcultivation is recommended two weeks after culture inoculation because most cultures with pure *Leptospira* were detected at this time point. In general, it has to be considered that *Leptospira interrogans* serovar Icterohaemorrhagiae strain RGA was used. The strain is well adapted to in vitro cultures and frequently used in the MAT. The strain might have characteristics that differentiate from a directly isolated serovar Icterohaemorrhagiae field strain, which might have influenced the study results.

According to our internal laboratory experience, filtration is efficiently used to remove contaminants from pure cultures with a high *Leptospira* density (about 10^8^ *Leptospira*/mL), which are used for the MAT. Filtration of urine samples with a 0.8/0.2 μm bacteria filter to remove contaminants is recommended in the literature [2]. In our study *Leptospira* were not detected in cultures from the filtered urine samples with a density of 10^6^ *Leptospira*/mL. Therefore, filtration of urine samples with a 0.8/0.2 μm bacteria filter to remove contaminants is not recommended. Urine samples with a *Leptospira* density of 10^6^ *Leptospira*/mL and lower are at risk of becoming false negative in culture due to the filtration. Nervig and Ellinghausen [25] reported successful cultivation of *Leptospira interrogans* serovar Grippotyphosa from swine urine after filtration (0.45 µm) but underline the limited number of *Leptospira* passing the membrane.

It is not possible to differentiate saprophytic from pathogenic *Leptospira* by microscopy. Furthermore, *Leptospira* change their morphological appearance, for example into spherical forms, depending on cell health [43]. Furthermore, the differentiation of *Leptospira* from other spirochaetal bacteria is not feasible. Molecular testing by qPCR makes it possible to both identify pathogenic *Leptospira* by targeting genes only present in pathogenic species, such as the membrane lipoprotein gene *lipL32* [44,45] and to measure *Leptospira* density both in culture and clinical samples [46]. Quantification is done by comparison to a defined standard and the growth of *Leptospira* in culture over a timespan can be demonstrated by comparing measurements at different time points. Compared to counting chambers, qPCR is less laborious to perform. Furthermore, by using only crude bacterial lysates obtained by heating culture supernatants as opposed to purifying leptospiral DNA by more expensive and tedious nucleic acid extraction procedures, we propose a quick and cost-effective way of estimating leptospiral copy numbers in cultures. Although we did not observe PCR-inhibition in this study, it is possible that contaminants present in crude lysates influence copy number estimation. Copy numbers reported in this study should therefore be interpreted as a means of comparing leptospiral loads within our experimental setting, rather than as absolute copy numbers. In our study, qPCR was used to verify the results of manual counting. Both methods showed a high correlation with two outlying cultures assigned to have few leptospires by manual counting but one showed a strong increase, whereas the other one strongly decreased in leptospiral copy numbers. This indicates that qPCR is well suited for confirming the presence and growth of *Leptospira* in culture. By using qPCR it was also possible to determine a statistically significant difference in leptospiral yield between EMJH and EMJH-STAFF cultures. 

Swine urine samples with pH > 7 yielded more cultures with detectable *Leptospira* than swine urine samples ≤7. Parker et al. [27] tested the influence of pH on *Leptospira interrogans* serovar Icterohaemorrhagiae in vitro and obtained comparable results. In our study, the timespan of 30 min between spiking of urine samples and inoculation to culture was sufficient for the urine pH to influence the success of *Leptospira* cultivation. In a therapeutic point of view, acidification of the urine of swine by feeding a specific diet might be effective to reduce the shedding of viable and therefore infectious *Leptospira* into the environment as described for other bacteria [47]. 

Densities as low as 10^4^ *Leptospira*/mL of *Leptospira interrogans* serovar Icterohaemorrhagiae in swine urine were detected in EMJH-STAFF as pure cultures after four weeks. It can be concluded that even if swine urine contains a density as low as 10^4^ *Leptospira*/mL isolation of *Leptospira* can be successful. The quantity of *Leptospira* shed by pigs is not sufficiently known. Barragan et al. [46] indicate 10^4^ to 10^6^ *Leptospira*/mL in the urine of cattle and deer. It is unknown if these quantities also apply to pigs. Urine samples with low contamination were selected, which might explain that many cultures were free of contaminants until week six. The reason why contamination appeared after six weeks in cultures from samples with high *Leptospira* density is inconclusive. A possible explanation is that *Leptospira* changed their morphology, for example because of a high culture density and lack of nutrients [43], which could have been confounded as bacterial contaminants.

Time of transport of swine urine samples to the laboratory and loss of *Leptospira* viability because of pH influence and growth of contaminants was assumed a critical point for successful *Leptospira* cultivation. In former studies *Leptospira* were isolated from swine samples after addition to a transport solution, consisting of culture medium or other nutrients, or processing samples within a few hours after sampling [16,19]. The mentioned transport solution is tedious to produce and frequently not available, especially for field veterinarians. PBS to alkalinize pH and dilute the urine [14] and a low temperature of 4 °C to reduce contaminant growth but not *Leptospira interrogans* serovar Icterohaemorrhagiae viability [48] was assumed to improve cultivation and isolation. PBS is cheap and easy to store and therefore a potential alternative transport solution for field veterinarians. However, no differences were observed between the evaluation results from urine samples stored with and without buffer for 24 h at 4 °C. Urine samples with an alkaline and acidic pH were used. According to the results from experiment one of the study, detection of *Leptospira* from samples with a pH ≤ 7 was reduced. The use of only acidic urine samples might show an effect of adding PBS on the detection of *Leptospira* in EMJH and EMJH-STAFF cultures. In conclusion, the addition of PBS to urine samples for transport can be recommended because *Leptospira* growth might be supported and no suppressing effect on *Leptospira* growth was found.

Few studies are available about the influence of sample material on successful *Leptospira* cultivation and isolation, especially regarding samples from swine [24,25,26]. The results of this study provide information about selected factors (selective agents, filtration, pH) influencing cultivation and isolation success of *Leptospira interrogans* serovar Icterohaemorrhagiae from swine urine. We also propose to verify results of microscopic examination of leptospiral growth by a quick and simple qPCR protocol. Further optimization of isolation techniques from different specimens, including understanding the environmental conditions needed for certain pathogenic *Leptospira* serovars to grow, is still necessary. A few recent studies are available [11] but there is still a lot of work to do to get a deeper insight into successful *Leptospira* isolation. A high diversity of pathogenic *Leptospira* with certain demands regarding their growth conditions is spread all over the world [2] and presumably, we do not know all of them. The aim for research and laboratory diagnostics should be to characterize circulating *Leptospira* serovars within a species and region and to better understand the epidemiology of the disease to efficiently prevent and control leptospirosis in animals and humans. 

## Figures and Tables

**Table 1:** Percentage (%) of ten EMJH-STAFF cultures from spiked swine urine samples with microscopically (dark field, 200×) detectable Leptospira without contamination at week one to seven weeks post inoculation.

	EMJH-STAFF Cultures: *Leptospira* without Contamination Detectable (%)						
	*Leptospira*/mL Culture						
week	1 × 10^5^	5 × 10^4^	1 × 10^4^	5 × 10^3^	1 × 10^3^	5 × 10^2^	1 × 10^2^
1	90	70	50	30	0	0	0
2	90	80	60	40	0	0	0
3	90	80	70	80	50	0	0
4	90	80	80	80	50	10	10
5	100	90	80	80	70	30	10
6	90	70	80	80	80	60	50
7	0	60	70	70	80	60	60

## Data Availability

Not applicable.
